# Time series big data: a survey on data stream frameworks, analysis and algorithms

**DOI:** 10.1186/s40537-023-00760-1

**Published:** 2023-05-28

**Authors:** Ana Almeida, Susana Brás, Susana Sargento, Filipe Cabral Pinto

**Affiliations:** 1 Instituto de Telecomunicações, Aveiro, Portugal; 2 Departamento de Eletrónica, Telecomunicações e Informática, Universidade de Aveiro, Aveiro, Portugal; 3 IEETA, DETI, LASI, Universidade de Aveiro, Aveiro, Portugal; 4 Altice Labs, Aveiro, Portugal

**Keywords:** Big data, Time series, Stream processing engines, Forecasting, Anomaly detection, Machine learning

## Abstract

Big data has a substantial role nowadays, and its importance has significantly increased over the last decade. Big data’s biggest advantages are providing knowledge, supporting the decision-making process, and improving the use of resources, services, and infrastructures. The potential of big data increases when we apply it in real-time by providing real-time analysis, predictions, and forecasts, among many other applications. Our goal with this article is to provide a viewpoint on how to build a system capable of processing big data in real-time, performing analysis, and applying algorithms. A system should be designed to handle vast amounts of data and provide valuable knowledge through analysis and algorithms. This article explores the current approaches and how they can be used for the real-time operations and predictions.

## Introduction

The concept of big data was mentioned for the first time in a paper published in 1997 [1]. The authors called the problem of dealing with large data sets, “the problem of big data”. These large data sets were characterized by not fitting in the main memory, making it challenging or even impossible to analyze and visualize them. Even 25 years later, most computers cannot load 100 GB to memory, let alone process it.

In the current era in which data is produced at high rates, information has a decisive role, and most computers cannot process vast amounts of data; thus, it was necessary to create new ways to process the data. These aspects were the big impulse for the appearance of big data technologies.

The first approach to deal with big data sets was to divide them into smaller segments. However, even then, the segments could be very large in most cases. Besides, few computers were able to make this type of processing. To tackle this issue, frameworks started to appear to deal with batches of data. Nevertheless, none of these approaches deals with one big problem: what can be done if the data set keeps growing, and data continues to be received over time? To answer this question, several frameworks that deal with data streams have appeared.

The main goals of using big data are: (1) predicting future events, and (2) gaining insights and discovering relationships; in multidimensional and large sample-sized datasets [2]. However, these goals bring challenges in terms of computation and methods.

Predicting future events is also known as forecasting. Forecasting tasks foresee dealing with time series data. Processing and analyzing time series data in real-time can be a game-changer for an organization. This article will focus on time series data. Three tasks stand out on the analysis and prediction of time series data: monitoring, forecasting, and anomaly detection. These tasks benefit from being executed in real-time. Moreover, these tasks can be applied to many contexts and use cases. Therefore, it is important to use a streaming framework to process data as it arrives.

Anomaly detection in data streams is beneficial and essential for organizations to detect problems before they achieve more significant dimensions: for instance, to notice an intrusion before the intruder can steal or damage data. Another example is to detect unexpected traffic congestion and activate the responsible authorities. Therefore, the anomaly prediction connected to time series data will also be dealt in this article.

Using data streams in different contexts allows us to extract knowledge and make decisions in real-time (or near real-time). This article will explore how we can deal with big data, particularly, time series big data. This article will also analyse which algorithms can be applied to data to make forecasts and detect anomalies.

The main contributions of this work can be summarized as follows:

- A comparative analysis of Stream Processing Engines (SPEs), including their characteristics and provenance, processing techniques, delivery of events, performance, and popularity.
- A discussion on forecasting algorithms, including statistical and Machine Learning (ML) algorithms, and the advantages and disadvantages of using each type of algorithm.
- A discussion on anomaly detection algorithms, the challenges of working with datasets containing anomalies, and the methods used to detect anomalies, such as statistical and ML approaches.

A comparative analysis of SPEs led us conclude that Spark is the most popular framework; however, Flink is better for data-intensive applications, and Heron scales better. Forecasting and anomaly detection methods bring value to organizations. While forecasting can allow better management of resources, anomaly detection can mitigate and eliminate problems. Regarding the type of methods used, statistical methods are usually lighter and more explainable, while machine learning methods are better when we have complex hidden patterns. The most recent published papers show a preference for deep learning techniques.

### Motivation

Working with huge amounts of streaming time series data can be a challenging task. With this in mind, we want to guide the reader on how this can be achieved. We will focus on three key relevant aspects:

1. **Stream processing frameworks:** these frameworks enable to process huge amounts of data, perform analysis, and apply algorithms in real-time.
2. **Forecasting algorithms:** these algorithms allow to predict future events. Therefore, they are essential for many organizations to perform informed decisions, manage resources, improve services, among others.
3. **Anomaly detection algorithms:** these algorithms allow to identify abnormal or unusual patterns. They can be early symptoms of something wrong, and we should be careful. They help us to improve security, quality, and efficiency.

Although the main focus of this work is the literature review on streaming frameworks, since we aim to work with time series data, we will also review the forecasting and anomaly detection algorithms; they play a crucial role in taking advantage of real-time processing capabilities. Therefore, with this survey, we aim to:

1. Identify the most relevant state-of-the-art regarding both data streams and algorithms.
2. Evaluate and compare different frameworks and methods to highlight each method or framework’s strengths, weaknesses, and limitations and when they should be applied.
3. Provide a guide for future research by identifying gaps in the current literature, areas that need further investigation, and other opportunities.

### Related work

This subsection provides an overview of other related surveys presented in the literature. Table 1 summarizes the subjects mentioned in the works presented in this article, both surveys and research works. In this section we will address the survey articles.

**Table 1:** Related work: summary

References	Big Data	Stream Processing	Real-time	ML & DL	Forecasting	Anomaly Detection	Survey	Research article	No. of papers
[3]	X	X	X				X		1
[4]	X	X	X					X	1
[5, 6]	X	X		X		X	X		2
[7]	X	X			X		X		1
[8–12]	X	X					X		5
[13]	X	X						X	1
[14]	X			X		X		X	1
[15, 16]	X			X			X		2
[17]	X					X	X		1
[2, 18–20]	X						X		4
[21, 22]		X	X	X		X		X	2
[23, 24]		X	X					X	2
[25]		X		X	X			X	1
[26]		X		X			X		1
[27–32]		X					X		6
[33–35]		X						X	3
[36, 37]			X	X		X		X	2
[38]				X	X	X		X	1
[39–44]				X	X		X		4
[45–53]				X	X			X	9
[54–57]				X		X	X		4
[58–77]				X		X		X	20
No. of papers	19	26	8	45	12	33	31	43	74

This article presents a literature review on how to process huge amounts of time series that are continuously being produced over time and need to be processed in real-time. Therefore, in Table 1, we consider papers regarding big data, stream processing, real-time processing, machine learning and deep learning, forecasting, and anomaly detection. In addition, we revised both surveys and research articles. Unfortunately, to the best of our knowledge, we did not find a paper analyzing all these topics. Nevertheless, we will compare our study with the most relevant works.

The most significant difference with work [9] regarding big data streams is that the authors of work [9] compared several tools, technologies, methods and techniques regarding data streams. However, we are more focused on data stream processing frameworks. In addition, the authors of [3] also discussed the concept of real-time associated with the processing of data streams, while the authors of [10] only perform a brief comparison of streaming processing frameworks. The authors of [10] conducted some practical evaluations of the streaming processing frameworks. Our survey presents a literature review. Similar to the work presented in [11], we are also researching progress in big data-oriented stream data mining; however, we focus on time series related problems, namely forecasting and anomaly detection.

### Article structure

The remainder of this article is organized as follows. "Big data stream processing frameworks" section is focused on big data and data stream processing frameworks. It starts by discussing the problem definition, followed by existing solutions, it presents the elaborations and a summary. This section characterizes big data and discusses its relationship with data streams, forecasting methods, and anomaly detection. We also present frameworks for processing data streams, compare them, and discuss some example cases where each one can be applied. Next, "Analysis and algorithms for streaming data" section discusses algorithms that can be applied in the context of big data, namely forecasting concepts and methods ("Time series forecasting" section) and anomaly detection strategies ("Anomaly detection" section). In this section, we focus on statistical, ML, and Deep Learning (DL) methods and their advantages and disadvantages. Each of these 2 sections presents a similar organization. Finally, "Conclusions and future research directions" section presents the conclusions and the challenges envisaged for future work, as well as some future research directions.

## Big data stream processing frameworks

### Problem definition

The evolution of traditional systems to streaming systems brings new processing and analysis capabilities and challenges. Firstly, we are no longer limited to bounded data, since we can process bounded and unbounded data. We are no longer required to divide or process data into multiple steps. Usually, a single step is enough. Besides, we no longer have to wait long periods for data to be processed. As we receive data, we process and obtain results and insights.

Designing the architecture of an application is an important task that should be well thought out. Considering that the streaming processing is part of an entire system, as a first step in the deployment of this component, the system requirements should be analyzed and task prioritization shall be evaluated. Choosing a SPE is not different. Some of the desired requirements that might be considered for real-time data stream processing are:

- Process large volumes of data;
- Integrate data from multiple data sources;
- Deal with data with different properties (multi-dimensional data, multiple entities, spatial-temporal dependencies);
- Deal with bounded and unbounded data streams;
- Deal with unsorted data, or delayed data;
- Detect data anomalies;
- Computation performance metrics (low latency, high throughput, high availability, high scalability).

As we stated before, the true value of big data comes from taking insights from the data and helping decision-makers. Therefore, efficient and precise algorithms implemented on scalable frameworks are needed to explore the data potentials. If we consider ML and DL in our analysis, we might add the model performance (error and training time) to the list. In the context of forecasting, metrics such as the Mean Squared Error (MSE) or $\hbox {R}^{{\textbf {2}}}$-Score can be useful [38]. In the case of anomaly detection, we may choose a high accuracy, high precision or even high recall method [16]. Since explanations play a crucial role in decision-making, the explainability of the ML model should also be considered [78].

There are several SPEs. Each SPE provides different features and has different properties. Moreover, each one can be more or less adequate according to the application.

#### Big data

**Fig. 1:**
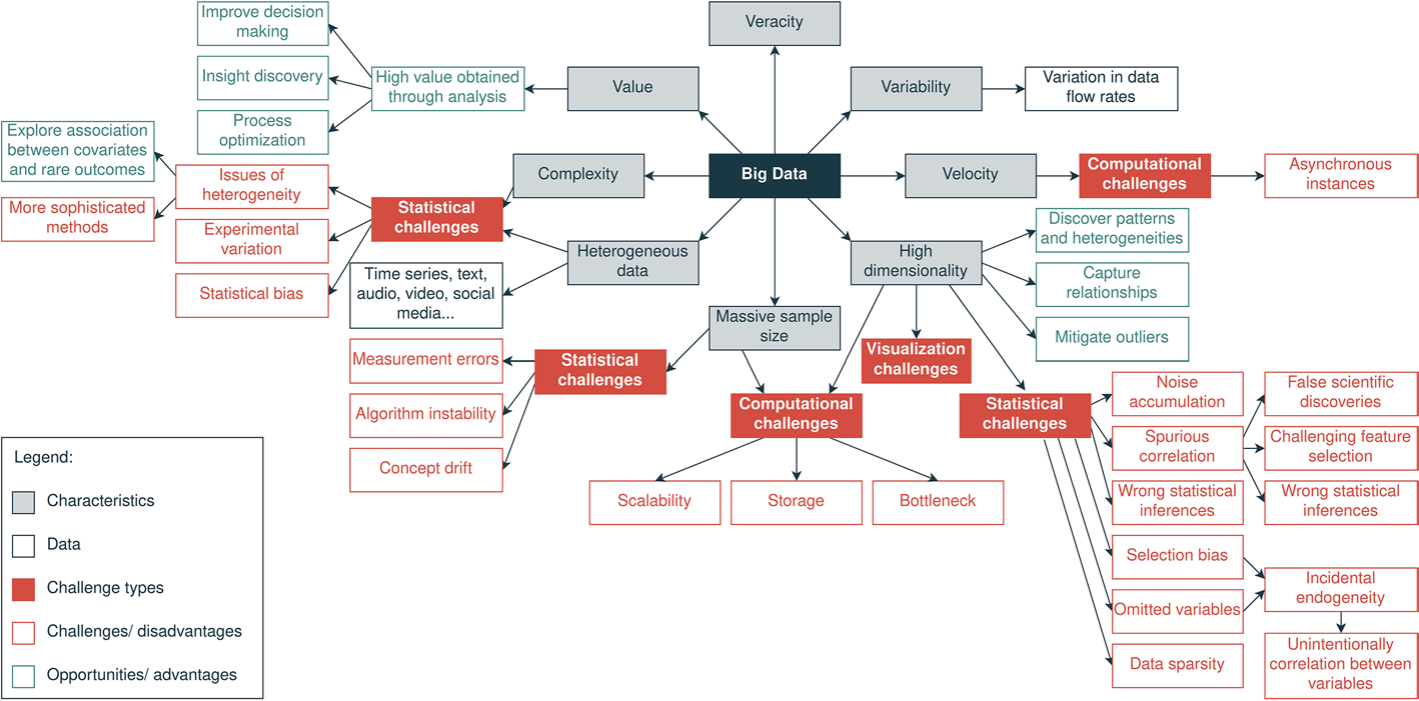
Big data taxonomy—information collected from [2, 5, 15, 17, 19]

The concept of big data has evolved through the years. First, big data started being depicted as a massive amount of data that does not fit in the main memory and requires more sophisticated ways of processing and visualizing [1]. This definition remains true; however, it is incomplete, since it is always being updated due to the data explosion [18] that occurred during the last decades. Defining big data is not a simple task because of its complexity. Figure 1 summarizes big data characteristics, challenges and opportunities.

As previously mentioned, this massive amount of data is characterized by massive sample size and high dimensionality [2]. Besides, data can arrive at high velocities and different flow rates [19]. Moreover, data can come from different sources [2], making it more complex. Data stream frameworks can receive data from multiple sources and process huge volumes of data, continuously arriving at high velocities. Several factors increase the complexity of dealing with big data, such as the variety of data that can be received [19]. For example, we can receive numerical values, text, images, sounds, video, or a combination of more than one type. In addition, our data can have a temporal component that brings additional complexity to the problem.

The maximum potential of big data is achieved when we trust the data and take advantage of it by analyzing it. Thus, we must identify inaccurate and uncertain data and deal with it [19]. In this context, the importance of anomaly detection methods is highlighted, especially the real-time detection of anomalies in data streams to mitigate anomalies as soon they happen.

Some of these characteristics bring statistical, computational, and visualization problems. For example, we can have algorithm instability, noise accumulation, spurious correlation, incidental endogeneity, and measurement errors regarding statistical problems [2]. On the other hand, regarding computation problems, we have storage, scalability, and bottleneck problems [2, 79]. Finally, visualization can be complex or even impossible when we have high-dimensional data.

Statistical problems can bring dangerous consequences, since they can lead to wrong statistical inferences or false scientific discoveries. For instance, an excellent example of a spurious correlation is the strong correlation (99.79%) between “US spending on science, space, and technology” and “Suicides by hanging, strangulation and suffocation” [80]. As we can understand, these two phenomena are unrelated. This is a well-known phenomenon in statistics, meaning that correlation does not imply causality. However, spurious correlations can go unnoticed depending on the context and the available knowledge.

To summarize, big data requires demanding computational resources, and its potential is unlocked through trust in data analysis. Therefore, several streaming frameworks emerged to process big amounts of data with low latency, high throughput, and high scalability. Furthermore, anomaly detection methods are essential in data streams [19], since they can suffer security attacks, have malfunctioning devices, or something unexpected may occur. We can also execute these methods in batch; however, when applied to real-time streaming data, they achieve their full potential. Besides, big data allows to (1) forecast future events, and (2) gain insights and discover relationships in data [2], both being important tasks, especially for decision-makers.

**Fig. 2:**
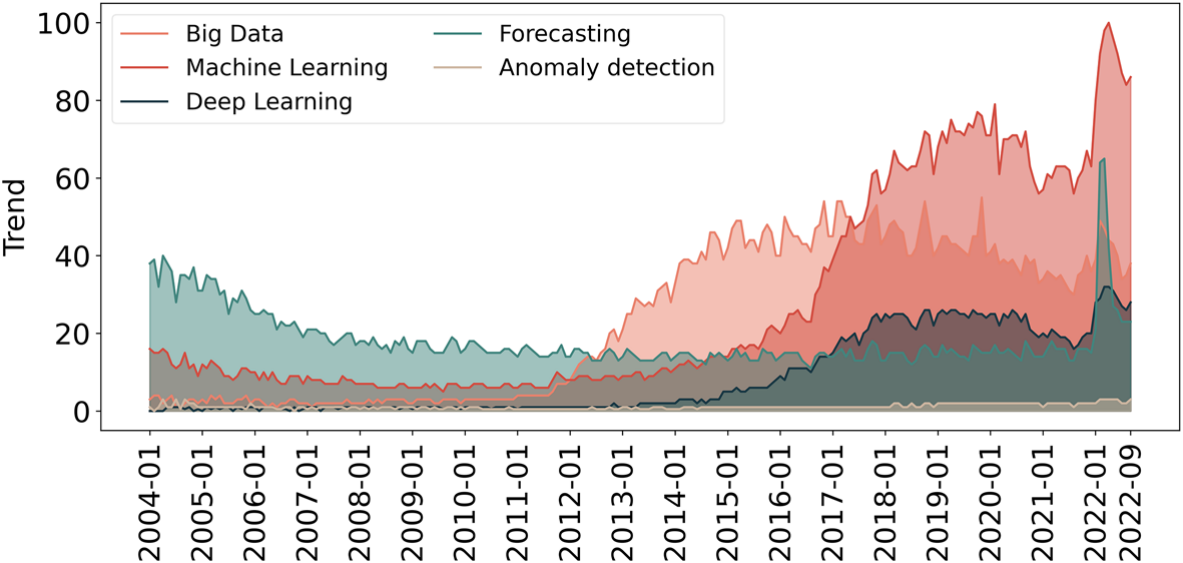
Google research trends over time—data collected from [81]

Big data analysis, forecasting, and anomaly detection are achieved through statistical, machine learning, or deep learning methods. Note that deep learning is a subset of machine learning. Figure 2 depicts Google searching trends through the years, by keywords. Big data, machine learning, and deep learning have a growing trend over the years. On the other hand, anomaly detection had a very soft increase. The searching trend forecasting decreases and reaches its peak in 2022; however, we can use other terms to express forecasting, such as prediction. Note that Google trends do not allow complex queries.

We can apply big data to a vast amount of scientific fields. We will present examples of use cases and applications for analyzing time series data streams in real-time. We will also include some examples that benefit from forecasting or anomaly detection methods.

In finances and economics, monitoring the stock market, detecting fraud, or forecasting the performance of assets, are high relevant tasks. In [25], the authors used Artificial Neural Network (ANNs) and data streams to forecast stock prices. Monika Arya et al. [21] proposed a real-time method to detect credit card fraud in data streams, using ANNs with ensemble trees.

Regarding health care and well-being, monitoring patients and having real-time processing capabilities can save lives. For instance, Leo Kobayashi et al. [82] created a patient monitor system using streams and multimodal data fusion. Their approach allowed them to analyse the data, conduct experiments and develop and apply algorithms. Another interesting application is to monitor and forecast the spread of infectious diseases. For instance, Ensheng Dong et al. [83] created an interactive dashboard to monitor COVID-19 using data streams.

We can also find works that benefit from using frameworks to process data streams in informatics and communications, such as monitor resource usage or detect security attacks. In [4], the authors propose an internet traffic monitoring system using streaming frameworks. And in [7], Liu et al. perform resource management and scheduling.

Other main areas with big data characteristics are smart cities and industry 4.0. One significant advantage is that they allow the creation of living labs, creating a space for learning and innovation. We can find several works to monitor and improve urban mobility, monitor water consumption and detect water leaks [84], and forecast traffic flow [38], among many others. Leonhard Hennig et al. [23] built a system to extract mobility and industry events from data streams. Qinglong Dai et al. [13] used a data stream framework with customized changes to process data from smart grids. Still, in the context of energy systems, Philsy Baban [24] could process and validate real-time streaming data. In [8], Sahal et al. discussed streaming frameworks and other tools to perform predictive maintenance for railway transportation and wind energy.

As can be observed, we can find big data applications in several different fields. Society can benefit greatly from big data; however, big data can also be dangerous. In this article, we will not explore the “dark side” of big data. For instance, it can serve for mass surveillance and persecution or increase the disparities among minorities. However, we hope that governments and institutions use big data for good. In this context, it emerged a new research area: “fair AI”, whose biggest goal is to combat racism, sexism, and other types of discrimination against minorities [85].

#### Real-time data stream processing

We use the term “big data” to define huge amounts of data [1] and the term “stream” to express data continuously being created and arriving [86]. This data can come from different sources and have different formats; its processing is not always trivial, especially if it is required in real-time.

**Fig. 3:**
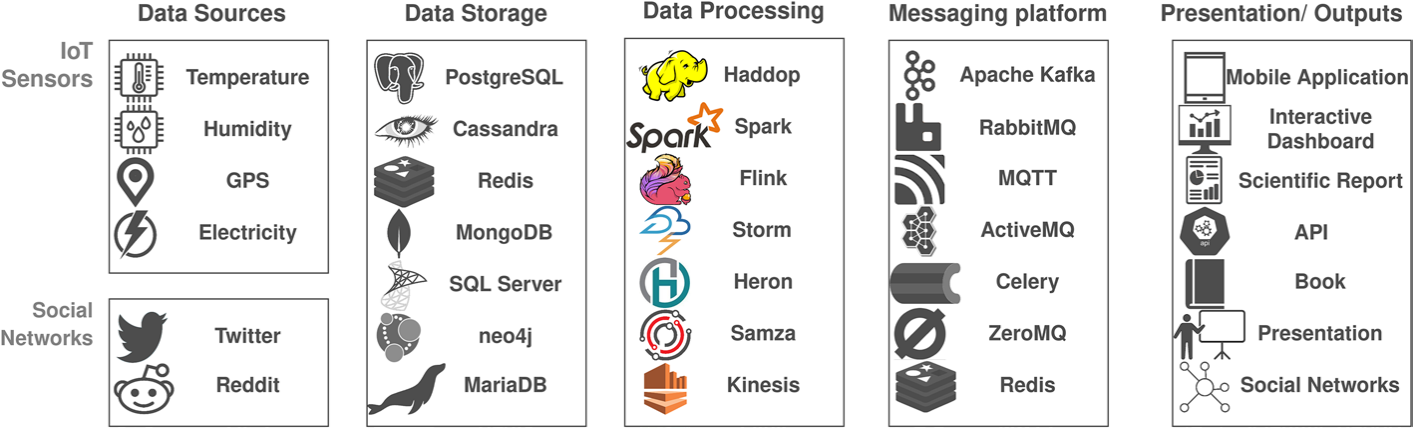
Big data applications components

Big data applications can have five types of components: data sources, a messaging platform, a processing module, a storage mechanism, and a presentation module. The data sources can be, among others, Internet of Things (IoT) sensors and social networks. These sources of information usually come from users, devices or activity logs. The messaging platform is responsible for sending data between modules. The processing module can be a streaming processing framework to ensure real-time processing capabilities. The storage mechanism can be a database or a data warehouse. Processed data can be presented in different ways, such as a web application, a mobile application, and a technical report. Figure 3 depicts the components of big data applications.

### Existing solutions

#### Fundamental concepts

In "Problem definition" we mentioned application requirements that can restrict the choice of a SPE. Now, we will discuss fundamental concepts that make it possible to have different data-processing techniques.

We may consider three types of processing: batch-based, stream-based, or event-based [87]. Batch processing is characterized by processing bounded data streams with a beginning and an ending. On the contrary, stream processing is characterized by the processing of unbounded data streams that do not have a known end. Besides, the data processing is performed as data arrives. If our application requires that we generate alerts or triggers if our data meets some conditions, we have event-based processing.

Concerning the processing model, we also have three types: at most once, at least once, and exactly once. At most once processing does not guarantee that the data is processed or persisted. In case of failure, we may have to deal with missing data. Usually, applications that choose at most once processing are more concerned with latency than reliability. On the contrary, at least once processing may process or persist duplicated data, but at least it guarantees that every data is processed or persisted at least one time. At last, exactly once processing just processes or persists data once.

**Fig. 4:**
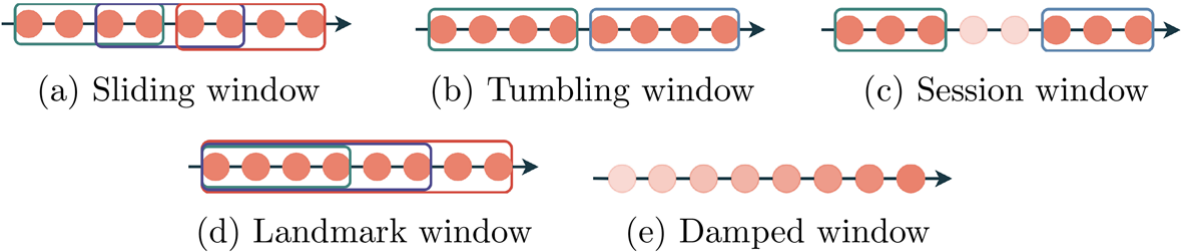
Processing window mechanisms

Window mechanisms specify how to divide the stream in order to aggregate time series data. There are six main processing techniques [26, 88]. The most basic mechanism is the single-pass in which we process each new sample only once. Several windowing mechanisms will be discussed. Nevertheless, a windowing mechanism can be defined as a function of the time or the number of events [27]. A sliding window mechanism is defined as a window with a fixed size that slides over the data stream [26]. Tumbling windows are non-overlapping sliding windows [88]. Session windows are similar to tumbling windows; however, in session windows, we have a gap between windows [88]. In a landmark window, it is specified a sample from which the window keeps growing [26]. This sample can be updated from time to time. At last, the damped window mechanism uses a fading mechanism in which, the most recent samples have a bigger weight, and, as time goes by, the samples loose their weight [26]. Figure 4 represents some of these window mechanisms.

Regarding stream-based processing, its methods can be considered stateless or stateful. If the processing is stateless, then the state is not preserved. We can use stateful processing if we want to know how many people buy a specific game per month. On the other hand, if the state is retained, the processing is stateful. This can be useful to measure how many people buy the game over time in a commulative maner.

#### Data processing frameworks

As aforementioned, we will discuss and compare different SPEs. We selected six SPEs: Apache Spark, Apache Flink, Apache Storm, Apache Heron, Apache Samza, and Amazon Kinesis. Besides, we decided to include Apache Hadoop for historical reasons.

Hadoop^1^ was the first framework that appeared to process large datasets using the MapReduce programming model. Hadoop is very scalable, since it can run on a single cluster, in a single machine, or spread on several clusters in multiple machines. Moreover, Hadoop takes advantage of distributed storage to improve performance by transmitting the code that processes the data instead of the data [89]. Besides, Hadoop provides high availability and high throughput. However, it can have efficiency problems when dealing with small files.

The major drawback of using Hadoop is that it does not support real-time stream processing. To deal with this problem, Apache Spark emerged. Spark^2^ is a framework for processing batch and streaming data, and allows distributed processing. According to Matei Zaharia [90], the creator of Spark, Spark was designed to respond to three big problems of Hadoop:

1. Avoid iterative algorithms that make several passes through the data;
2. Allow real-time streaming;
3. Allow interactive queries.

Instead of MapReduce, Spark uses Resilient Distributed Datasets (RDDs) that are fault-tolerant and can be processed in parallel. Spark also provides scalability, and since its early releases, it has proved to outperform Hadoop [33]. Spark is helpful for data science related projects. Besides its main component, Spark provides several libraries for Exploratory Data Analysis (EDA), ML, graph analysis, stream processing and SQL analytics.

Two years later, Apache Flink^3^ and Apache Storm^4^ were created. While Spark uses micro-batches for stream processing, Flink and Storm can perform stream processing natively. Flink can process batch and streaming data. In Flink, we can process streams with specific temporal requirements. For example, we may consider processing or event time. In case of event time, Flink allows to deal with delayed events. Besides, Flink provides watermark support, allowing a trade-off between latency and completeness of data. Storm and Flink are similar frameworks, generating some discussion regarding their differences [91] and which of the following stand out:

1. Storm only allows stream processing;
2. They both can perform stream processing with low latency;
3. The API offered by Flink is more high-level and provides more functionalities;
4. They have different strategies to provide fault tolerance (Storm employs record-level acknowledgements while Flink uses a snapshot algorithm).

Storm is a good streaming framework; however, its capabilities to scale are not enough for more demanding applications. Besides this, debugging and managing Storm can be complex tasks. In this context, Apache Heron^5^ emerges, as the successor of Storm. A paper published in 2015 [34] announced this transition at Twitter.

Apache Samza^6^ is a framework that provides real-time processing, event-based applications, and *Extract, Transform and Load* capabilities. Samza provides several APIs and presents an architecture similar to Hadoop, but instead of using MapReduce, it has the Samza API, and it uses Kafka instead of the *Hadoop Distributed File System*.

Finally, Amazon Kinesis^7^ is the only framework presented in this article that does not belong to the Apache Software Foundation. Kinesis is actually a set of four frameworks instead of a data stream framework. In this work, we refer to Amazon Kinesis to talk about the *Kinesis Data Streams* framework to simplify. Kinesis can easily be integrated with Flink.

### Elaboration

The processing frameworks present different properties, which makes it challenging to choose one framework without understanding the differences. Therefore, we should choose the framework that suits best our use case.

Firstly, we decided to look at the nature of each framework. Although several frameworks belong to the Apache ecosystem, most were not created by Apache. They were later integrated into the Apache family through The Apache Incubator.^8^ Table 2 resumes the nature of each one of them.

**Table 2:** Frameworks characteristics and provenance

Framework	Initial release	Creator	Incubation year	Type of software
Apache Hadoop	2006	Apache Software Foundation	N.A.	Free and open source
Apache Spark	2009	University of California	2013	
Apache Flink	2011	Apache Software Foundation	2014	
Apache Storm	2011	Backtype	2013	
Apache Heron	2014	Twitter	2017	
Apache Samza	2013	Linkedin	2013	
Amazon Kinesis	2013	Amazon	N.A.	

Table 3 contains information about the processing techniques available (batch or stream) and the delivery of events (at most once, at least once, exactly once). As we already mentioned, Hadoop only provides batch processing. Storm and Heron only provide stream processing. All other frameworks offer both batch and stream processing. However, Spark provides stream processing through micro-batches. Regarding the delivery of events, most frameworks guarantee that the events are processed exactly once or at least once. Heron offers three types of delivery, the two mentioned above and at most once. Besides, these frameworks provide drivers for several programming languages, the most popular are Python and Java.

**Table 3:** Framework processing techniques and delivery of events

Framework	Processing	Delivery of events
Apache Hadoop	Batch	N.A.
Apache Spark	Batch and Stream (micro-batch)	Exactly once
Apache Flink	Batch and Stream	Exactly once
Apache Storm	Stream	At least once
Apache Heron	Stream	At most once, at least once, exactly once
Apache Samza	Batch and Stream	At least once
Amazon Kinesis	Batch and Stream	At-least once

Performance-wise, some experiments have been conducted to compare the different SPEs. Note that it is difficult to make a fair comparison due to the lack of experiments that contemplate all frameworks. Therefore, we started by a performance comparison regarding the frameworks. This comparison considers the information available in the official documentation of each framework, which is present in Table 4. One of the most important characteristics when choosing a framework is the ability to process information in real-time. However, there needs to be a consensual definition of what real-time means. Gomes et al. [3] focused their study on this concept in the context of data streams and big data. According to the authors, there are different intents when discussing real-time. For example, real-time could mean an immediate response. Another possibility is the guarantee of low latency: some consider the time the system should answer, while others refer the time the system must answer. For a more fair comparison, in this discussion, we will focus on real-time as the property of having low latency.

**Table 4:** Performance comparation

Framework	Latency	Throughput	Scalability	Fault tolerance
Apache Hadoop	High	High	High	Replication in the HDFS
Apache Spark	Low	High	High	Resilient Distributed Dataset
Apache Flink	Low	High	High	Incremental checkpointing (uses markers)
Apache Storm	Low	High	High	Record-level acknowledgements
Apache Heron	Low	High	High	High fault tolerance
Apache Samza	Low	High	High	Host-affinity, and incremental checkpointing
Amazon Kinesis	Low	High	High	High fault tolerance

Most of these frameworks present low latency, which is good when we are processing significant amounts of data and want to process it in real-time. Hadoop is the only one that is considered to have high latency. All frameworks present high throughput and high scalability. However, Hadoop only allows scaling vertically. Regarding fault tolerance mechanisms, all frameworks deal with fault tolerance.

After this initial study, we look for works that compare some of these frameworks to make an unbiased comparison. In 2015, Namiot et al. [10] made an introductory comparison of the properties of Storm, Spark, Samza, Apache Flume, Apache Kafka, Amazon Kinesis, and IBM InfoSphere.

Besides the noticeable differences between Hadoop and Spark, Pooja Choudhary et al. [28] conducted some experiments to compare these two frameworks. They concluded that Spark uses more memory than Hadoop, needing less execution time. However, the authors of [35] mentioned that Spark might not be the best framework if our application requires low latency and high throughput.

The authors of [29] compared the performance of Spark, Flink, and Storm under saturation conditions (the maximum streaming load that the frameworks could support without delay). This comparison is insightful if we want to choose the best framework for a data-intensive application. Flink presented the highest saturation level, while Storm had the worst CPU usage. Even when failure recovery mechanisms are activated, Storm performance decreases by 50%, while Flink only decreases 10%. Nevertheless, Spark can surpass Flink if we are not concerned with latency.

Inoubli et al. [12] performed experiments in which they compared Spark, Storm, Flink, and Samza. They observed that Spark achieved the worst processing rates compared to the other three frameworks. Flink and Samza were more efficient, especially when messages had a more considerable size. Flink CPU usage was lower; however, Flink could outperform Storm if the CPU consumption allowed was increased. Spark requires more RAM, less disk access, it is slower in processing messages, and uses less bandwidth.

In 2019, in the context of a smart city, Hamid Nasiri et al. [30] evaluated three different frameworks: Spark, Flink and Storm. They started by fixing the input rate and compared the performance with two nodes versus eight nodes. With two nodes, Flink presented the lowest latency and the highest throughput. Flink delivered a similar performance with a slightly higher throughput with eight nodes. The improvements on Spark and Storm were more significant, but Flink was still the best. On the other hand, Spark had the worst latency. With eight nodes, Spark presented a similar throughput to Flink; however, it reached the highest throughput peaks. They analyzed the impact of changing the input rate and the number of worker nodes. We can conclude that the performance of Flink is similar to Storm, even when using no acknowledgements in Storm. The most significant difference is the throughput in which Flink is better than Storm; however, Storm seems to scale better, and with eight nodes, Spark is the best of them all in terms of throughput. At last, they measured CPU and network utilization. Flink achieved the lowest CPU utilization and the highest network utilization. Storm and Spark achieved similar performances.

Kolajo et al. [9] compared 19 tools and technologies for data streaming; however, only half of them supported both batch and streaming processing. On another work [31], in 2019, the authors compared the performance of five stream processing systems: Storm, Flink, Spark, Kafka Stream, and Hazelcast Jet. Storm has the best memory consumption, and presents good stability. Flink presents the lowest latency. Spark presents the highest throughput and has a good compatibility with ML libraries.

In 2020, LinkedIn published a post [92] showing some improvements performed on Samza. These improvements provided Samza with more considerable throughput capabilities when compared with Flink.

Later in 2021, Krzysztof Wecel et al. [32] selected six frameworks, but has chosen to focus their analysis on comparing Spark and Flink. They concluded that Spark is more memory efficient while Flink is more CPU efficient. The authors also mentioned that, while performing their experiment, they found a problem that led to delays in the implementation phase: missing detailed documentation. We were already aware of this problem, especially with Flink.

Heron brings an extensive set of advantages to users that want to transit from Storm to a more scalable framework. The API available for Heron is compatible with the one available for Storm. Heron requires fewer resources (less CPU usage) and provides performance improvements (more throughput and less latency). Currently, Heron is in the incubating phase at The Apache Incubator [93].

To understand the frameworks popularity, we decided to perform two experiments using Scopus.^9^ These experiments were performed on August 9th, 2022. In the first experiment, we try to understand the popularity of the different frameworks over the years. In the second experiment, we try to perceive how many publications exist when we consider different criteria.

For the first experiment, we created three queries. The example below contains the queries for the Apache Hadoop framework. Similar queries were performed for the remaining frameworks.

**Q1**: apache w/ hadoop
**Q2**: TITLE-ABS-KEY (apache w/ hadoop)
**Q3**: TITLE-ABS-KEY (apache w/ hadoop) AND (LIMIT-TO (SUBJAREA,“COMP”) OR LIMIT-TO (SUBJAREA,“ENGI”))

Firstly, we perform a general search using only the framework’s name. Secondly, we restrict papers with the framework’s name in the title, abstract or keywords. Lastly, we limit the subject area to papers published in the engineering field or computer science.

**Fig. 5:**
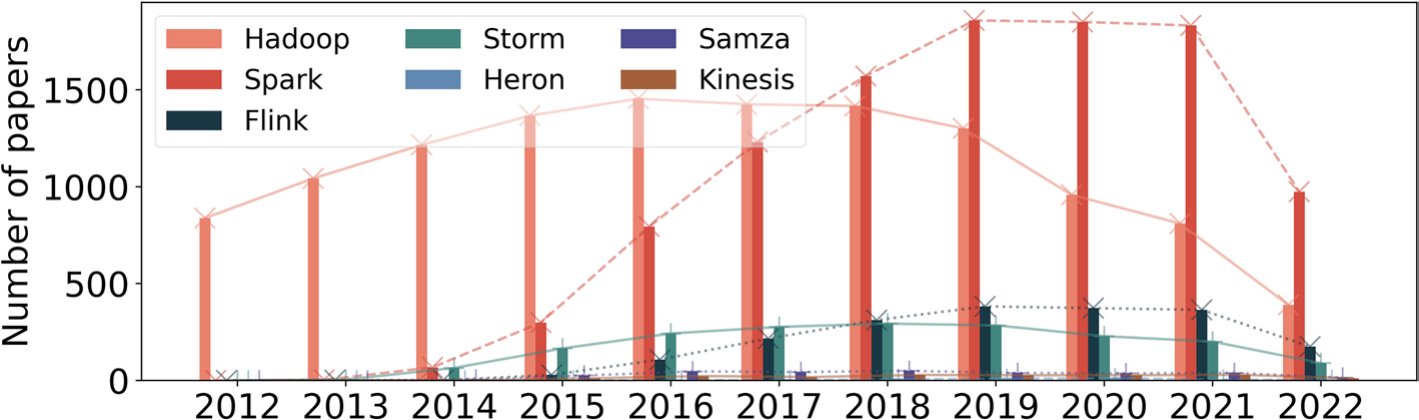
Data processing frameworks: Popularity over the years first query

Figure 5 contains the results of the first query. We can visualize that Hadoop is the dominant framework in the first years. This happens because Hadoop is the oldest, and most frameworks did not exist or did not belong to the Apache Software Foundation at the time. The most popular streaming framework is Spark. Following Spark, the popularity of Flink and Storm is similar. Finally, Heron, Samza and Kinesis are the most unpopular frameworks.

**Fig. 6:**
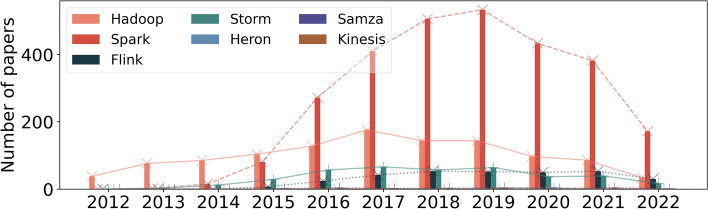
Data processing frameworks: Popularity over the years second query

Figure 6 presents the results of the second query. When we restrict papers with the framework’s name in the title, abstract or keywords, we can visualize that Spark is the dominant framework. This might indicate that most papers that mention Hadoop only mention it because it was the first relevant framework. Another explanation is that Hadoop is the framework used in the study, but was not the subject of the study. Therefore, this second query is more focused on studying the framework, not its usage.

**Fig. 7:**
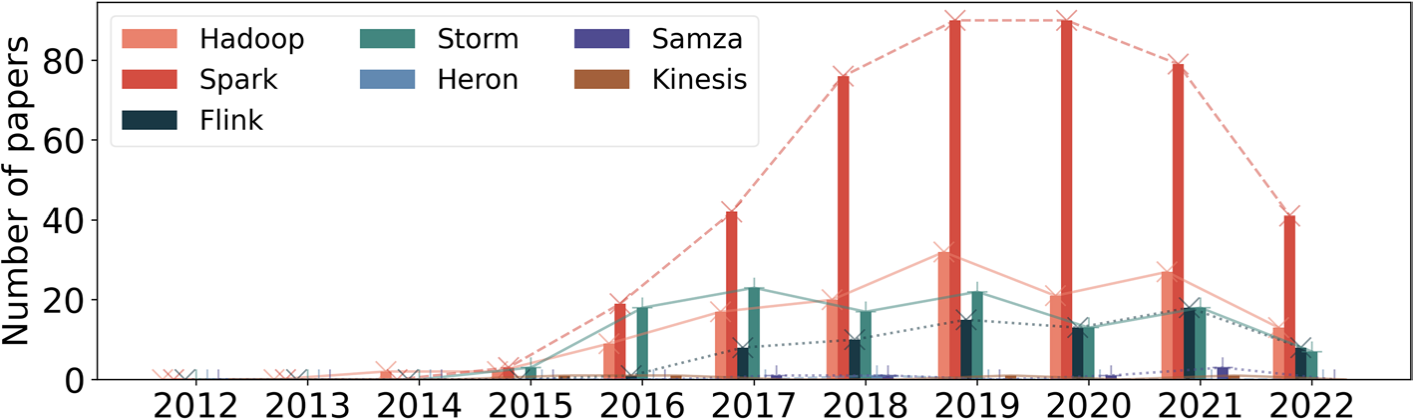
Data processing frameworks: Popularity over the years third query

What we can visualize in Fig. 6 is intensified in Fig. 7 when we limit the subject area. Figure 7 shows the results of the third query.

In the second experiment, we evaluate the number of papers that considered stream-related concepts and algorithms. Our goal is to understand, for instance, how many articles that addressed forecasting also addressed streams. We started with two basic queries. First, query 4 helps to understand how many papers contain the word forecast or other words derivated from the word forecast, such as forecasting or forecasts. Query 5 helps to understand how many papers include anomaly detection or outlier detection. Query 6 is an additional query to understand how many papers also include ML or DL.

**Q4**: forecast*
**Q5**: (anomaly w/ detection) OR (outlier w/ detection)
**Q6**: (machine w/ learning) OR (deep w/ learning)

**Fig. 8:**
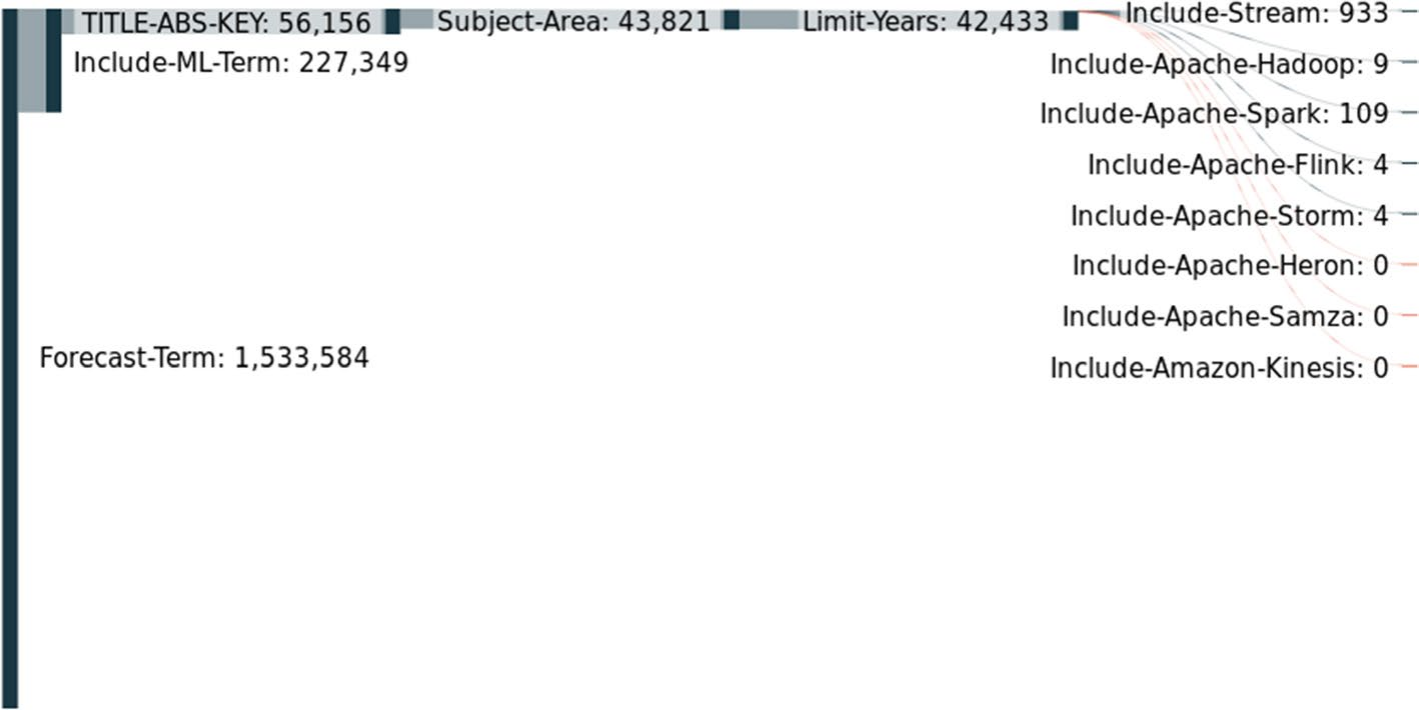
Forecast versus Stream

Figure 8 contains the results for forecasting terms. We start by performing query 4, and we named forecast-term. Then, we also included query 6, which we called ML-term. Then, we selected only the papers that had both terms in the title, abstract, or keywords. The next step was to limit by subject area (as in the first experiment). Then, we limited the search by the years from 2012 until 2023. Finally, we included different terms in order to answer our initial question. We separated the terms stream and the several frameworks. As we can visualize, we started with 1.5 million papers, and in the end, only 1 thousand had terms related to streams.

**Fig. 9:**
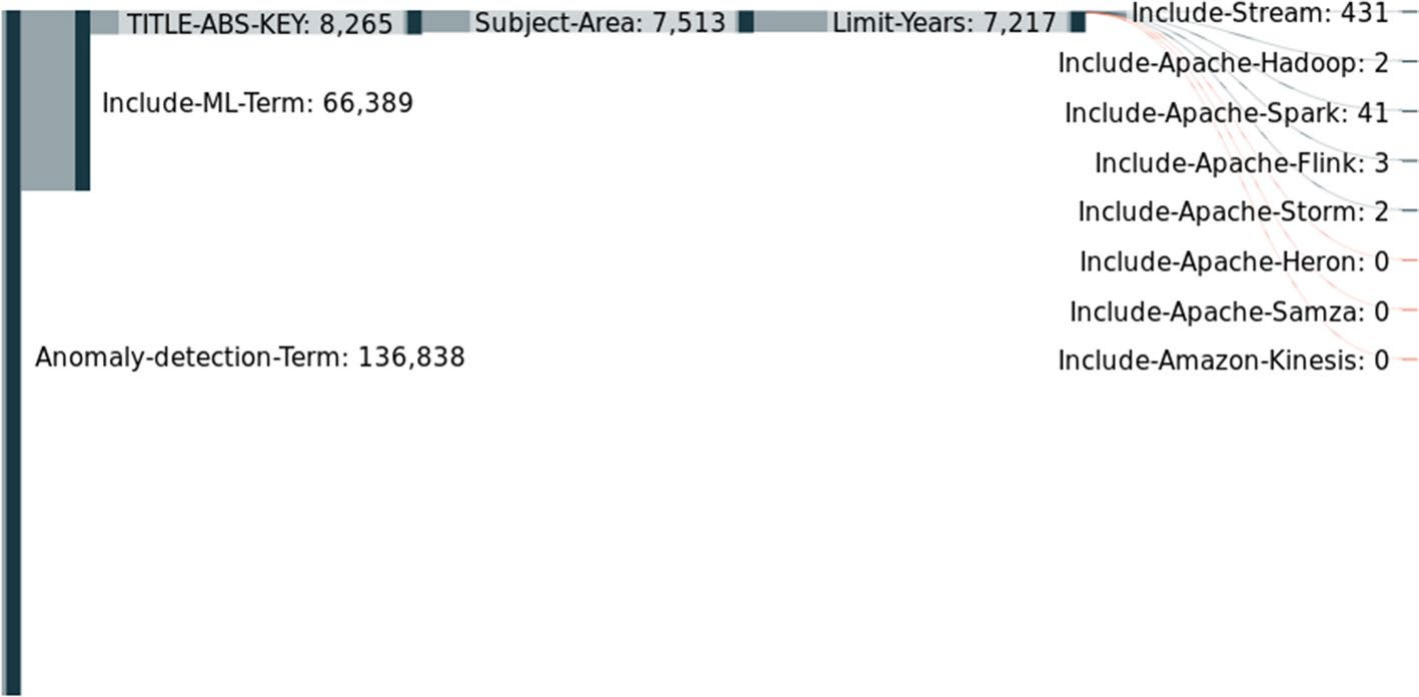
Anomaly detection versus Stream

Figure 9 contains the results for anomaly detection. The only thing that changed with Fig. 5 was the initial term that, in this case, was the anomaly detection term, query 5. As we can visualize, we began with 136 thousand papers, and in the end, only five hundred had terms related to streams.

Only a few papers consider streaming and forecasting concepts because a forecasting algorithm, to provide the most benefits, should perform real-time forecasting. Moreover, given the complexity of implementing a stream-based forecasting system and a forecasting algorithm, researchers can be more focused on developing one of these tasks when they publish their work. The same can be applied to anomaly detection concepts and other applications.

### Summary

Choosing the best SPE is a critical engineering task that should consider the following. Foremost, only Spark, Flink, Samza and Kinesis allow both batch and stream processing. In addition, Spark and Flink do not allow missing or repeated data. However, Heron enables the choice of any delivery. Flink is the best framework for data-intensive applications, presenting the lowest latency and highest throughput. However, Storm seems to scale better. Recent studies have proven that Samza has a better throughput than Flink, and Heron scales better than Storm. Nevertheless, Spark and Storm are the most popular stream frameworks. Heron is a good substitute for Storm, allowing Storm users to transition easily.

## Analysis and algorithms for streaming data

In the scope of ML, several tasks can take advantage of streaming technologies, such as regression, classification, clustering, forecasting, anomaly detection, and frequent pattern mining.

In this section, we decided to focus on two tasks related with time series: forecasting ("Time series forecasting" secrtion) and anomaly detection ("Anomaly detection" section).

### Time series forecasting

#### Problem definition

Humans are constantly trying to predict the future. Millions of years ago, when we started counting time, we also began to make predictions. One of the questions that most hunt humanity, and that several societies, religions and individuals tried to guest, is when doomsday will occur. Several dates have been proposed over the years, but until now, none of them has been correct.

Forecasting is a prediction task in which we try to predict future events accurately. To make good forecasts, we should understand the phenomenon and the causes that influence the phenomenon. We can use historical data, events that may occur, and other information that may contribute to the forecasting task [94]. For example, when we look at the sky and see dark clouds, we can (most certainly) guess it will rain.

Accordingly, with the domain of our problem, we should look for data other than the phenomenon’s data. For instance, Wasiat Khan et al. [45] used data from social media and financial news to predict the stock market’s performance. However, the authors recognize that not all stocks are influenced the same way. Besides, the authors noticed that some stocks were more influenced by social media news, while others were more influenced by financial news. Ahmad Ali et al. [46] considered the spatial-temporal dependencies and several temporal patterns (current, daily, and weekly) to predict crow flows. The use of external factors, such as weather conditions, holidays, and events was also crucial in this context.

Forecasting tasks can be classified as short, medium or long-term forecasts [94]. These terms are used if the forecast is made for the near future, medium future or distant future. For instance, we may want to predict how many people will travel to a tourist destination in the next hour, in the next week, or in the next year.

Usually, short-term forecasting is only relevant in a short interval. Therefore, we might benefit from performing the forecasting in real-time or near-real-time. On the other hand, medium and long-term forecasting is not needed immediately; therefore, we can perform them offline.

Forecasting problems use time series data. A time series is the evolution of one variable (or more) over time. A time series is a stochastic process, time-indexed, thus making statistical properties relevant. When we only have one variable, we have a univariate time series. We have a multivariate time series when we have more than one variable. Usually, when we are in the presence of a univariate time series, we call it a time series [94–96].

**Fig. 10:**
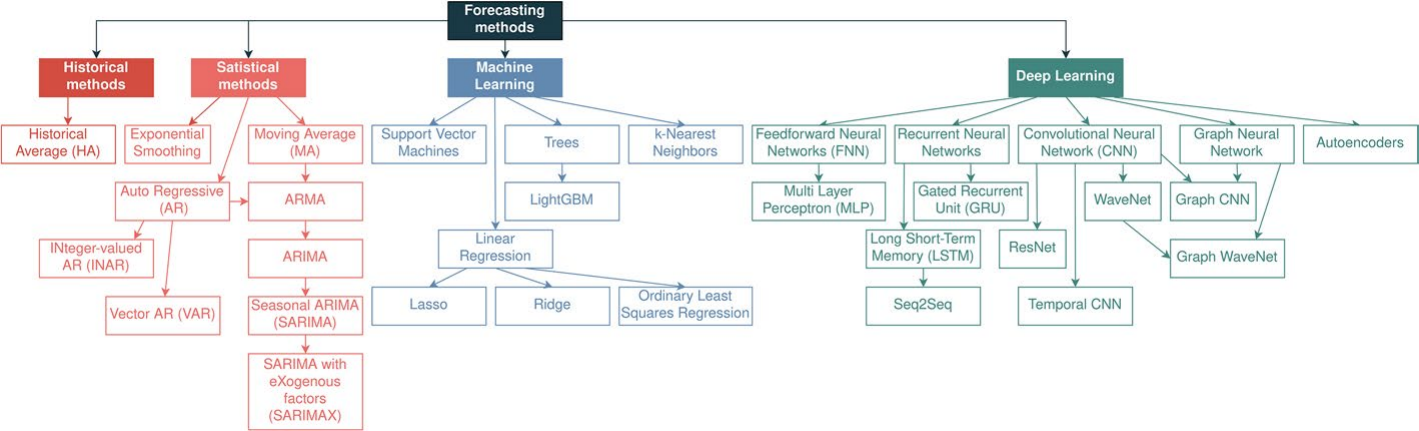
Forecasting methods

Time series data is similar to streaming data, since we can look at the data arriving from the streaming with a temporal component and a sequential order. However, this does not mean that all data from streams are time series, even though they might have a timestamp associated.

#### Existing solutions

There are three types of forecasting methods: historical, statistical, and ML. Historical methods only look at past values to forecast new ones. The most popular historical method is the Historical Average (HA), which can be found in the literature [47], especially as a baseline. Statistical methods are mainly based on the Auto Regressive (AR) method. They are also considered usually as a baseline. For instance, we can find Auto Regressive Integrated Moving Average (ARIMA) in work [47]. ML approaches, particularly DL, have been highlighted more recently, and several novelty methods have been proposed.

We can find forecasting works related to energy consumption and pricing. Bangzhu Zhu et al. [48] used an SVM-based method with mixture kernels to forecast carbon prices. Razak Olu-Ajayi et al. [49] predicted the energy consumption of buildings using ML and DL models, and concluded that ANNs are more suitable to make predictions. In [50], Zhang et al. proposed a Multi-view Ensemble Learning Model (MELM) to forecast traffic of base stations to save power in cellular networks. Their multi-view methods had four views: a temporal, a spatial, one dedicated to events, and the last view for residual information. For the temporal component, they analyzed the auto-correlation, the trend, and the seasonality of the data, and they used the Seasonal Auto Regressive Integrated Moving Average (SARIMA) to perform short and long-term forecasting. They used a spreading model based on a grid system to observe and capture the spatial dependencies. The authors observed that different regions have a different number of users, and they observed mobility transferring from nearby regions. They used a decision tree to capture the influence of events, since they cause changes in traffic. They considered four types of events (holidays, weather, concerts, and news). For the residual information, they used a top-k regression tree.

Another explored topic is related to traffic. To predict the flow of crowds, in [51] it is proposed a framework called Forecasting Citywide Crowd Flows (FCCF). The authors used human mobility data, weather conditions, and road network data. First, they divided the human mobility data into two edge flow categories: inflow and outflow. Besides that, they split the region into small regions. Then, they decomposed the flows into seasonal, trend, and residual and built a model for each one of the flows. For the seasonal and trend components, they created an Intrinsic Guassian Markov-Random-Field (IGMRF) for each component. For the residual, they explored the spatiotemporal dependence and built a spatiotemporal residual model that uses a Bayesian network. Then, the models were aggregated to give the final prediction.

The authors in [52] proposed a multi-view network model called Deep Multi-View Spatial-Temporal Network (DMVST-NET). They observed that, in most cases, including a region that presents a weak correlation with the region we want to predict decreases the model’s performance. Usually, distant regions are less correlated, but this is not always true. Considering this all, the authors chose to create three views: a view for the temporal component, another for the spatial component (they only consider nearby regions), and the last one for semantic relations (the regions are far away but present similar demands). They used a Long Short-Term Memory (LSTM) for the temporal component, a Convolutional Neural Network (CNN) for the spatial component, and a Graph Neural Network (GNN) to capture the semantic relations.

In [53], the Multi-Task Learning Temporal Convolutional Neural Network (MTLTCNN) method is proposed for short-term passenger demand prediction. The authors started by using a Spatio-Temporal Dynamic Time Warping (ST-DTW) algorithm to select the most relevant features. The proposed method is multi-task, having one task per region. Each task comprises a Temporal Convolutional Neural Network (TCNN), and the tasks share information between them, namely spatiotemporal correlations. Ahmad Ali et al. [46] proposed an ANN model based on graphs and convolution to predict crowd flows. In addition, they explored spatiotemporal dependencies and external factors. The authors of [47] proposed an architecture that uses graphs, convolution, and recurrency to forecast traffic. Their approach explores spatiotemporal dependencies.

In 2018, Spyros Makridakis et al. [39] published the results of the fourth edition of a forecasting accuracy competition. This competition discouraged the submission of complicated ML models that required high computational capabilities. Most of the best methods were combinations of statistical models. One of the best methods was a hybrid ML (using Recurrent Neural Network (RNN)) and a statistical approach (exponential smoothing). Unfortunately, some of the submitted methods were based only on ML and achieved the worst results. Later in 2021, Spyros Makridakis et al. [40] published the results of the fifth edition of the forecasting accuracy competition. The goal was to predict the sales of a retail company represented by 42.840 time series. Most of the competitors used LightGBM-based methods, a ML method based on trees. In the top five, the first two top methods were essentially a weighted combination of LightGBM models, the third winner was a weighted combination of a Neural Network (NN), the fourth place was a non-recursive LightGBM, and the fifth was a recursive LightGBM.

A literature review on deep learning methods for financial time series forecasting [43] presented eight methods commonly used: Deep Multi Layer Perceptron (DMLPs), RNNs, LSTMs, CNNs, Restricted Boltzman Machines (RBMs), Deep Belief Networks (DBNs), Autoencoders (AEs), and Deep Reinforcement Learning (DRL). The authors highlight the preference of researchers in using RNNs, specially LSTMs, with financial data. However, as the authors identified, CNNs and Graph-based networks still need to be explored when using financial data. Meanwhile, Masini et al. [44] reviewed both ML and DL methods for financial forecasting; their main focus was NN, regression trees, bagging, and regression. The authors emphasized the use of ML models (including DL models) in the presence of large datasets.

Table 5 resumes the revised works. In this comparison, we did not include the survey articles. As we can visualize, different approaches emerged over the last years for both ML and DL methods. Most of the authors used more than one metric to compare the methods.

**Table 5:** Comparison of forecasting methods

TM	Method	Year	PM	MV	Metrics	Notes
ML	MELM, based on SARIMA, and on a top-K regression tree method	2017	[50]	Yes	ARMSE	
	FCCF, based on Bayes Networks and Gaussian Markov random fields	2016	[51]	Yes	RMSE	
	Based on LSSVM, and mixture kernels	2022	[48]	Yes	MSPE, MAPE, RMSE	
	Weighted combination of LightGBM models	2021	[40]	Yes	WRMSSE	The best method of the M5 competition.
DL	DMVST-Net, based on LSTMs and CNNs	2018	[52]	Yes	MAPE, RMSE	The authors included a view for semantics.
	Hybrid approach of exponential smoothing with RNNs.	2018	[39]	Yes	sMAPE	The best method of the M4 competition.
	MTL-TCNN, based on Temporal Convolution, Convolution, and DTW	2020	[53]	Yes	MAE, MAPE, RMSE	
	Based on GCN and GRU	2021	[47]	Yes	MAE, MAPE, MSE	Drawbacks: The complexity of the method.
	Deep Neural Network	2022	[49]	Yes	$\hbox {R}^{2}$, MAE, RMSE, MSE	Practical comparison of methods
	Based on CNNs, graphs, and LSTMs	2022	[46]	Yes	RMSE, MAPE	

$^{\textrm{TM}}$ Type of model

$$^{\textrm{PM}}$$ Proposed Method

$$^{\textrm{MV}}$$ Multivariate

#### Elaboration

Figure 10 contains some of the methods used in forecast tasks. Forecasting may be accomplished using statistical methods or DL-based methods. Both approaches have advantages and disadvantages. Depending on the context, statistical methods may be more advantageous than DL methods and vice-versa. While statistical methods are explainable, they are usually more robust in short-time predictions, and they present the best results in short-time contexts. They are usually not suitable for long-term forecasting.

ANNs present some disadvantages. The first problem is to find the weights of the inputs. The training process will update the model weights in each iteration; however, the optimization algorithm used may not lead to the minimum error or loss and can lead to overfitting. The training process can be extensive, making its adoption difficult in some contexts. ANNs also require a lot of information and great computational power when compared with statistical methods.

One of the big problems with ML algorithms is the lack of transparency, especially in ANNs. ANNs are often seen as “black boxes” [41]. In order to solve this issue, a new topic has emerged in the scope of ML: explainable models. Explainability plays a crucial role in the understanding of a particular problem. A correct prediction is not always enough, since it can have real impacts in terms of security, ethics, mismatched objectives, privacy, and others [42].

The more relevant advantage of using DL based methods is the possibility of working with multidimensional data, in some cases exploring the relationships between space, time, and other factors that may influence the prediction. Statistical methods may be more beneficial regarding forecasting methods with real-time stream processing, since they are lighter. However, we should consider the application requirements, the data, and the threshold between execution time and other performance metrics.

**Fig. 11:**
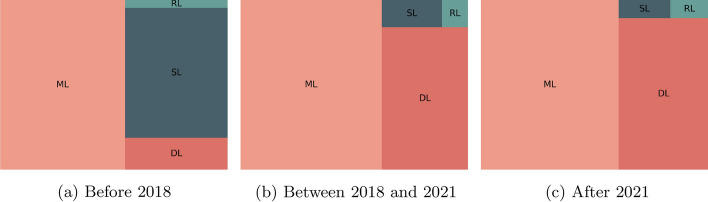
Evolution of the popularity of type of methods regarding forecasting over the years. ML stands for Machine Learning, DL for Deep Learning, SL for Statistical Learning, and RL for Reinforcement Learning

We decided to compare the type of methods used in forecasting in terms of popularity over the years, highlighting the last years. Figure 11 contains the relationship between the number of documents retrieved from Scopus when we perform the query example Q7. As we can observe, the use of machine learning and deep learning for forecasting increased over the last few years.

**Q7:** TITLE-ABS-KEY ( forecasting AND ( “machine learning” OR “ml”) )

**Fig. 12:**
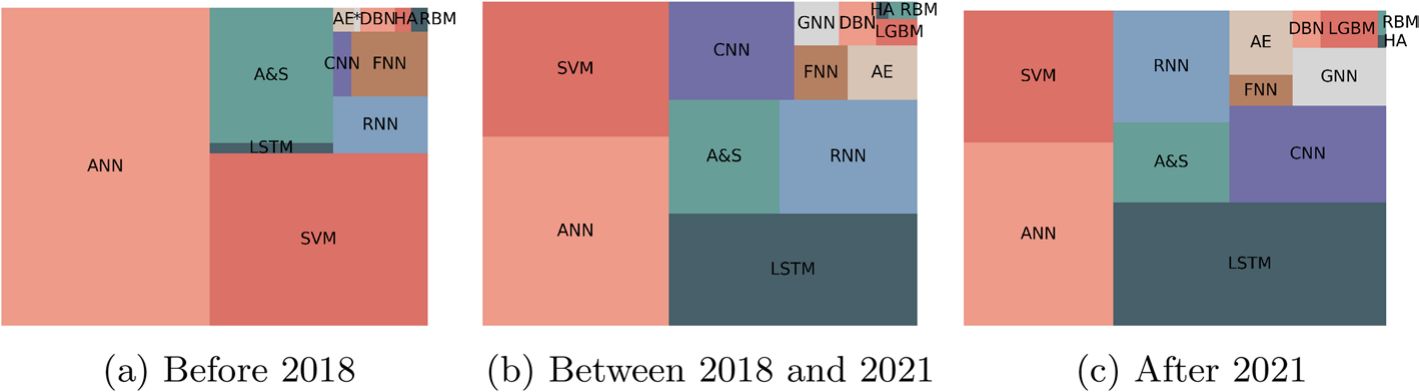
Evolution of the popularity of methods regarding forecasting over the years. ANN stands for Artificial Neural Network, SVM for Support Vector Machine, LSTM for Long Short-Term Memory, A &S for ARIMA and SARIMA, RNN for Recurrent Neural Network, CNN for Convolution Neural Network, FNN for Feedforward Neural Network, AE for Autoencoder, GNN for Graph Neural Network, DBN for Deep Belief Network, LGBM for LightGBM, HA for Historical Average and RBM for Restricted Boltzmann Machines

We also compared the methods used. Figure 12 contains the obtained results. Before 2018, the type of methods that were more mentioned were the ANNs. This can happen for two reasons: it was used the generic architecture of ANN, or the authors used the word when referring to a specific type of ANN. For instance, a LSTM is a type of ANN. Over the years, we can observe an increase in the use of LSTMs, CNN, RNNs, AE, and GNNs. The popularity of Deep Learning methods does not mean that the statistical ones are not important. It just reflects the evolution and trends of research methods.

#### Summary

Forecasting is an essential task when working with time series datasets. We can have different forecasting horizons, such as short, medium, and long-term. We can apply this type of method to different contexts and use cases.

Classical methods are mainly based on Auto-Regression. Regarding machine learning methods, LightGBM proved to be efficient. In the case of deep learning methods, the most used are based on LSTMs, CNNs, AEs, and GNNs. As we discussed, all methods have their positive and negative aspects. In addition, the application and intent of the problem can make the choice of the technique easier to select.

### Anomaly detection

#### Problem definition

An anomaly occurs when something unexpected happens. We can observe anomalies in our daily lives, for instance, a cold day (as if it were winter) in the middle of the summer. We can visualize the anomalies in data. If we look for the chart that contains the daily temperatures measured in the summer, we would see an anomalous point in relation to the other points. However, not all anomalies are expressed in the same way. Anomalies can be classified by their nature, they can be a point anomaly, a contextual anomaly, or a collective anomaly [54].

A point anomaly can be identified when we compare it with the rest of the data [55]. Remembering the “cold day in the middle of the summer” example, if we only had data from the summer, we would have a point anomaly if the observed temperature was very different from all others.

A contextual anomaly happens in a particular context [55]. If we had data from the entire year, we would observe that in the winter there are low temperatures. The point is anomalous because it happens in the summer and not in the winter. This is similar to a conditional anomaly, which depends on the context to be classified as an anomaly.

A collective anomaly is a collection of points that are considered anomalous when compared with the remaining dataset [56]. They can be, for instance, an abrupt change in the temperature of the summer. Another example would be a day in which it is verified a smaller variation of temperatures. As we know, temperatures are higher in the summer. However, we can have fluctuation throughout the day. From the examples above, we can conclude that anomalies can also be present in time series, and can be isolated outliers or abrupt changes.

There are several challenges associated with the detection of anomalies. Anomalies are not always known or noticeable, and it is difficult to define what may be considered as anomalous. Besides that, there is always some noise associated with the anomaly detection. As an example, network attacks can change, evolve, and adapt, marking this as a complex problem, and allowing negative impacts to happen from the presence of false negatives and false positives in the analysis [54, 57].

#### Existing solutions

Anomalies are known for being rare in datasets. It is because of that property that they are considered anomalies. In a dataset containing anomalies, and if our goal is to identify them, we will have a class imbalance problem. This problem is amplified when dealing with big data. There are three different techniques to solve this issue [16]:

- Data-based techniques: using sampling methods, we can reduce the level of imbalance;
- Algorithm-based techniques: we can reduce the bias towards the majority group;
- Hybrid techniques.

Learners can have difficulties identifying anomalies, especially in highly imbalanced datasets, such as decision trees and logistic regression [16]. Moreover, some classification metrics are more sensitive to imbalanced classes. Regarding the evaluation metrics, some metrics are highly affected and are not recommended, such as accuracy and error rate. Other metrics, such as precision, and recall, can be used, but they alone are usually not enough [16]. The *F*-measure metric is a weighted average of precision and recall and is highly used in this context.

To detect anomalies, statistical learning approaches can be used. In [58], Hochenbaum et al. used seasonal decomposing to extract the trend and the seasonal components. They proposed two techniques: the seasonal Extreme Studentized Deviate (ESD), and the seasonal hybrid ESD, which adds the median and the Median Absolute Deviation.

Some methods to detect anomalies are signal-based. In [59], the authors could effectively detect sharp increases in the local variance using wavelet filters and pseudo-spline filters. In [97], Muñoz et al. used correlation-based techniques.

Principal Component Analysis (PCA) based approaches were explored in [60, 61]. In [60], the authors applied wavelet transformations to network traffic data. Then, it is applied PCA to extract the nature of anomalies. Finally, they use a mapping function to detect the anomalies. In [62], the authors could also localize the source of anomalies by incorporating the network structure information with the PCA model. They used the Karhunen Loève Expansion to get spatial and temporal correlations. In [61], the authors proposed the use of Minimum Covariance Determinant (MCD) with Robust Principal Component Analysis (rPCA). As PCA might have issues associated with introducing the outliers in the subspace, rPCA tackles it, with a computational cost. The use of MCD helps to ease the computational cost.

We can also find in the literature approaches based on the k-Nearest Neighbors (KNN) algorithm. In [63], the authors proposed a Transductive Confidence Machine (TCM) with KNN for online anomaly detection. They could improve their results by applying instance selection. The authors of [22] compared Naive Bayes, Support Vector Machine (SVM), and decision trees, and in [36] it is used Naive Bayes.

**Fig. 13:**
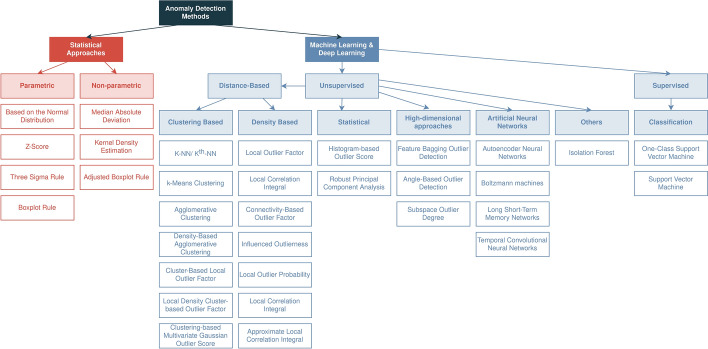
Anomaly detection methods

Several works are based on ANNs, such as [37, 64–74]. In [64], motivated by the presence of a high rate of false alarms and improving accuracy, Hussain et al. proposed a FeedForward Neural Network (FNN) to detect anomalies in cellular networks. They accomplished high accuracy and a low False Positive Rate (FPR), proving the usefulness of FNNs. The work in [65] used a LSTM to detect network attacks through the anomalies present in data. They tested two types of baselines. In the first one, they only used cleaned data to train the model (without anomalies). In the second one, they used dirty data to train the model (with anomalies). They concluded that the dirty baseline models achieved the best results, which is good when no completely clean dataset exists. In [66], it is proposed the Parallel Subagging-GRU-based network (PSB-GRU)Parallel Subagging-GRU-based network (PSB-GRU) method. The model uses a Gated Recurrent Unit (GRU) network for long-term dependencies, a genetic algorithm to optimize the training process, the Spark platform to improve train efficiency, and subagging smoothly to improve the model’s generalization.

In [67], it is compared the performance of several RNN-based methods. The authors concluded that LSTM networks achieve the best results in terms of performance; however, the other RNN-based network also achieved good results. The works in [65–67] allow to conclude that sequential NN are suitable to detect anomalies. In [68], it is proposed a CNN-based method to extract spatio-temporal and other features from data with a threshold-based separation method to detect anomalies. The architecture had four convolutional layers. They achieved good results; however, they recognize that they need a more lightweight method to perform online anomaly detection. The authors of [74] also used a CNN. They were able to achieve better performance, in some cases, in architectures with one convolutional layer when compared with two or three convolution layers. However, their methods did not outperform RNN-based methods. The authors of [69] explored how CNNs can fail. The authors concluded that a one-pixel attack can mislead CNN-based networks. Increasing the number of layers (three convolution and three pooling layers) and retraining contributes to a more robust detection.

The authors of [70] proposed an ensemble method based on RBM and SVM. They tested their method in real time and achieved good performance. The work in [71] used Self-Organizing-Maps (SOM). Their model is computationally light, presenting results with a very low delay. In [37] the authors also use SOM with *k*-*medoids*, and they perform a two-step clustering. They achieved fast online detection and a multistage decision to distinguish different anomalies. In [72] it is proposed an autoencoder-based method with convolution. The use of autoencoders allowed the authors to capture non-linear correlations between features. The use of convolution has also reduced the training time. In [73], stacked autoencoders are used with a one-class classification model. The use of autoencoders allows the selection of the most relevant features and the reduction of data dimensionality.

Other approaches, such as the one proposed by [75, 76] are tensor-based. A tensor is a structure similar to a multidimensional array with three or more dimensions. When we have one dimension, we have a vector (denoted as a first-order tensor), and if we have two dimensions, we have a matrix (second-order tensor) [76]. In [75], the proposed method is based on tensor decomposition. The method in [76] is based on tensor factorization, and we have a two-phase anomaly detection. Tensor-based methods are useful when we have complex data with high-dimensional orders.

Table 6 resumes the revised works for anomaly detection. We can visualize different types of methods. In anomaly detection, one of the most important tasks is the fair evaluation of the methods. Usually, in an anomaly detection problem, we have the class imbalance problem, as mentioned above. To compare better the evaluation metrics used, we decided to create Table 7. False Positive Rate, True Positive Rate, and accuracy are the most frequently used metrics. The class imbalance highly affects the accuracy, and this metric should not be used, especially without other metrics.

**Table 6:** Comparison of anomaly detection methods in literature

Type of Method		Method	PM	Year	Metrics
Statistical		Based on recursive least squares, and sparsity maximization	[75]	2016	F-Score, ROC, Residual error
		Based on wavelet filters and pseudo-spline filters	[59]	2002	TP
		Based on correlation techniques	[97]	2016	Absolute error
		Based on Dirichlet process	[77]	2019	Accuracy, FPR, TPR
		Based on seasonal decomposition and robust statistical metrics	[58]	2017	F-Score, TPR, Precision
ML	Based on PCA	Based on rPCA	[61]	2017	FPR, FNR
		Based on PCA and the Karhunen Loève Expansion	[62]	2013	AUC, ROC
		Based on multi-scale analysis, PCA, and wavelet transforms	[60]	2015	ROC
	Based on KNN and TCM		[63]	2009	FPR, TPR
	Naive Bayes		[36]	2018	Accuracy
	Based on SVM	SVM	[22]	2015	Accuracy
		Based on RBM and SVM	[70]	2019	Accuracy, FPR, F-Score, ROC, Precision
	Based on SOM	SOM	[71]	2005	FPR, TPR
		SOM with k-medoids	[37]	2018	FPR
	Based on tensor factorization		[76]	2017	FPR, TPR
DL	Based on FNNs		[64]	2019	Accuracy, Error rate, FPR, F1-Score, Precision, TPR
	Based on RNNs	Based on GRU	[66]	2021	Accuracy, F1-Score, Precision, TPR
		Based on RNNs	[67]	2017	Accuracy, AUC, FPR, Loss, ROC, TPR
		Based on LSTM	[65]	2018	AUC, ROC
	Based on CNNs	Based on CNNs	[68]	2018	TPR
		Based on CNNs	[74]	2018	MCC
		Based on CNNs and FNNs	[57]	2018	Accuracy, FPR, TPR
		Based on CNNs	[69]*	2020	Accuracy
	Based on Autoencoders	Based on Autoencoders and convolution	[72]	2018	Accuracy, FPR, ROC
		Based on Stacked Autoencoders	[73]	2019	Accuracy

$$^{\textrm{PM}}$$ Proposed Method

**Table 7:** Evaluation metrics used in anomaly detection problems

Metrics	Papers	# of Papers
Absolute error	[97]	1
Accuracy	[22, 36, 57, 64, 66, 67, 69, 70, 72, 73, 77]	11
AUC	[62, 65, 67]	3
Error rate	[64]	1
FPR	[37, 57, 61, 63, 64, 67, 70–72, 76, 77]	11
FNR	[61]	1
F-Score	[58, 70, 75]	3
F1-Score	[64, 66]	2
Loss	[67]	1
MCC	[74]	1
Precision	[58, 64, 66, 70]	4
Residual error	[75]	1
ROC	[60, 62, 65, 67, 70, 72, 75]	7
TP	[59]	1
TPR, sensitivity or recall	[57, 58, 63, 64, 66–68, 71, 76, 77]	10
# of distinct papers		25

#### Elaboration

Figure 13 contains some methods used in anomaly detection. Traditional statistical methods can fail in the face of big data and data with several dimensions. On the other side, ML methods can deal with high dimensionality. Supervised methods achieve good performance in detecting anomalies [6]. However, they have problems detecting new unseen types of anomalies. Unsupervised methods are good at detecting new anomalies [14].

**Fig. 14:**
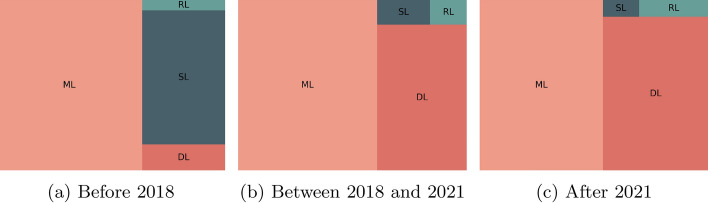
Evolution of the popularity of type of methods regarding anomaly detection over the years. ML stands for Machine Learning, DL for Deep Learning, SL for Statistical Learning, and RL for Reinforcement Learning

**Fig. 15:**
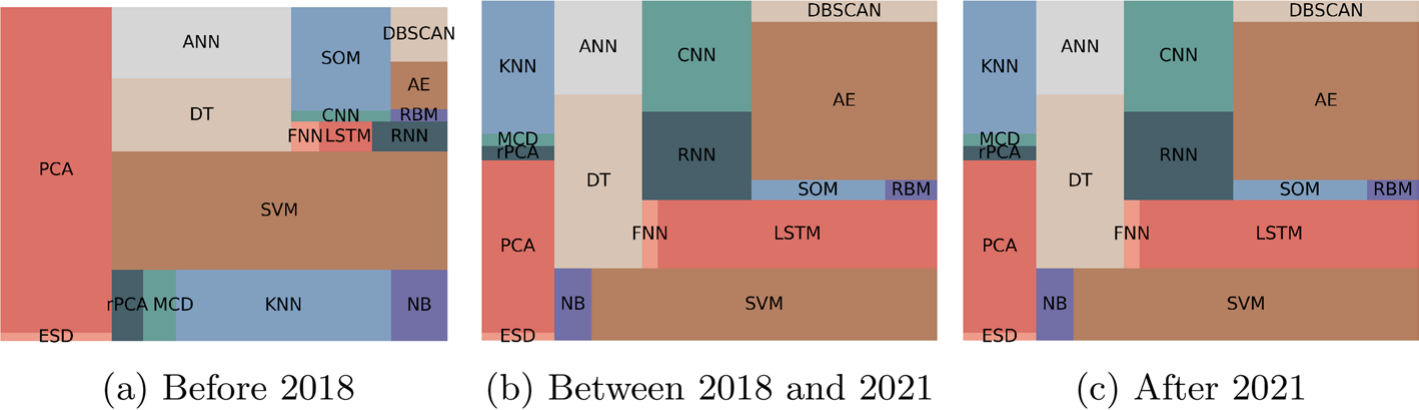
Evolution of the popularity of methods regarding anomaly detection over the years. ESD stands for Extreme Studentized Deviate, PCA for Principal Component Analysis, rPCA for Robust Principal Component Analysis, MCD for Minimum Covariance Determinant, KNN for k-Nearest Neighbors, NB for Naive Bayes, SVM for Support Vector Machine, DT for Decision Trees (and includes random forest), ANN for Artificial Neural Network, FNN for Feedforward Neural Network, LSTM for Long Short-Term Memory, RNN for Recurrent Neural Network, CNN for Convolution Neural Network, SOM for Self-Organizing-Maps, RBM for Restricted Boltzmann Machines, AE for Autoencoder and DBSCAN for Density-Based Spatial Clustering of Applications with Noise

Figure 14 contains the evolution of the popularity of the type of anomaly detection methods over the last few years. The use of statistical methods decreased while the use of deep learning methods increased. Currently, most of the published works use machine learning and deep learning. Similarly, Fig. 15 contains the evolution of the popularity of techniques over the last few years. As we can observe, methods such as PCA, SVM, and KNN lost popularity over time, while the focus evolved to the use of CNNs, RNNs, LSTMs and AE.

#### Summary

As can be concluded from the above information, there are several methods that can be applied to anomaly detection. Regardless of the chosen method, we must take into consideration some problems associated with the nature of the data. The first class of problems that the methods can be vulnerable to are data poisoning attacks. In this context, a data poisoning attack might be something that we consider normal, being abnormal in the training phase. In [77], the authors deal with this problem by separating the training phase from the learning process.

Different methods should be considered when dealing with anomalies in data streams, since there is not one single method able to detect all types of anomalies. Furthermore, data streams are very susceptible to data poisoning attacks, since the use of supervised methods does not know the most recent data and needs to be regularly updated. Moreover, we should evaluate, once more, the threshold between execution time and other performance metrics. Finally, in the context of big data and ML, we should take into account that we are dealing with a class imbalance problem.

## Conclusions and future research directions

Data by itself can have no value for organizations and society. However, we can transform data into knowledge and improve decision-making through analysis. Nevertheless, dealing with big data can be a complex problem, especially when the data keeps growing over time. In this context, Stream Processing Engines emerged. They are an essential tool for processing big data in real-time. In this work, we presented some frameworks to process data streams in real-time, and we compared them. Spark is not a native streaming framework since it uses micro-batches, which brings some performance issues. However, Spark is the most popular framework with several exploratory data analysis and machine learning modules. On the other side, Flink can deal better with data-intensive applications, while Heron seems to scale better.

We also presented approaches to deal with common big data problems, such as forecasting and anomaly detection in real-time. Applying these algorithms in real time can be very beneficial for organizations. For instance, the use of forecasting can help organizations to optimize the use of services and resources. On the other side, using anomaly detection algorithms can prevent or minimize problems before they happen, such as network attacks. Finally, we discussed statistical, machine learning, and deep learning approaches. Statistical methods are more explainable and computationally lighter. On the other side, machine learning methods deal better with complex data and can predict longer times.

As future research directions, we would like to suggest real-time analytics and algorithms over big data time series streams. Namely, having time series related machine learning and deep learning algorithms take advantage of online learning for providing real-time analysis, forecasts, and anomaly detection. Another possible research direction is the development of explainable methods focused on time-series.

## Data Availability

Not applicable.

## Footnotes

1 https://hadoop.apache.org/.

2 https://spark.apache.org/.

3 https://flink.apache.org/.

4 https://storm.apache.org/.

5 https://heron.apache.org/.

6 https://samza.apache.org/.

7 https://aws.amazon.com/kinesis/.

8 https://incubator.apache.org/.

9 www.scopus.com.
